# Exploring the Genetic Foundations of Salt Tolerance in Common Vetch (*Vicia sativa* L.) via Genome-Wide Association Analysis

**DOI:** 10.3390/genes17010032

**Published:** 2025-12-30

**Authors:** Hui Jin, Jumei Zhang, Yordan Dimtrov, Xue Yang, Ruonan Du, Yu-e Wu, Danna Chang, Rui Zhang, Haibin Zhao

**Affiliations:** 1 Institute of Forage and Grassland Sciences, Heilongjiang Academy of Agricultural Sciences, Harbin 150086, China; jinhuicaas@126.com (H.J.); zjm312@aliyun.com (J.Z.); ydtsvetkov@163.com (Y.D.); yxflax@126.com (X.Y.); drn0713@126.com (R.D.); lunawye@163.com (Y.-e.W.); zr0705@126.com (R.Z.); 2 Institute of Agricultural Resources and Regional Planning, Chinese Academy of Agricultural Sciences, Beijing 100081, China; changdanna@caas.cn

**Keywords:** association analysis, germination, marker-assisted selection (MAS), common vetch, salt tolerance

## Abstract

Background/Objectives: Common vetch (*Vicia sativa* L.) is a globally cultivated leguminous crop, valued for its high nutritional content and role in sustainable agriculture. Methods: To identify loci or genes significantly associated with salt tolerance, we conducted a genome-wide association study (GWAS) using 172 common vetch accessions primarily from diverse geographic regions. Single-nucleotide polymorphisms (SNPs) were obtained through re-sequencing, and five salt tolerance-related traits, including the germination rate (GR), germination potential (GP), germination index (GI), shoot length (SL), and root length (RL), were evaluated under salt stress conditions. We have identified 20 loci significantly associated with salt tolerance-related traits, and explaining 9.7–21.8% of the phenotypic variation. Notably, 13 loci exhibited pleiotropic effects on multiple traits; include *qST1.1* (associated with SL, GR, GI), *qST1.3* (RL, SL, GP), *qST2.5* (SL, GR, GI, GP), and *qST2.7* (SL, RL, GP, GI), and should be prioritized in future breeding programs. All 20 loci are novel compared to previous reports. Furthermore, we identified 7 candidate genes encoding key regulatory proteins, including a zinc finger MYM-type protein, ubiquitin-like domain-containing protein, transcription factor bHLH, ethylene-responsive transcription factor, auxin-responsive protein, and serine/threonine-protein kinase, as potential regulators of salt tolerance. Conclusions: This study advances our understanding of the genetic basis of salt tolerance in common vetch and provides valuable loci, molecular tools, and elite accessions. HZMC1352, GLF303, GLF301, HZMC1387, GLF306, GLF368, GLF342, HZMC1384, HZMC1355, GLF307, HZMC1366 are used for improving salt tolerance in breeding programs.

## 1. Introduction

Common vetch (*Vicia sativa* L.) is a widely cultivated and versatile legume crop valued as green manure, forage, and food [1,2,3,4]. Soil salinization poses a major challenge to global agriculture, severely limiting crop growth and yield. Nearly 20% of the world’s farmland and 50% of irrigated areas are affected by soil salinity, which threatens the sustainable development of global agriculture [3,4]. Under salt stress, plant growth-related traits, such as seed germination, plant height and root length are significantly reduced [3,4,5]. Consequently, breeding salt-tolerant crops is one of the most effective and sustainable ways to utilize saline farmland [5,6,7,8]. As green manure, common vetch exhibits strong nitrogen-fixing capacity with dry biomass with 3.57% N, 0.32% P, and 2.68% K. Incorporating common vetch into soil can reduce synthetic fertilizer use approximately 30%. Long-term application could significantly enhance the soil organic matter content and its accumulation rate [9,10,11]. As forage, the dry hay of common vetch contains 177.0–219.0 g/kg crude protein, 9.6 MJ/kg metabolizable energy, and shows an in vitro organic matter digestibility of 727.8 g/kg [6,12]. Additionally, common vetch seeds could be used as food, with a higher content of amylose content (46.7%) and a milling yield of 30%, and surpassing that of field pea. The seeds of common vetch are rich in resistant starch, contributing to a low glycemic index, making them ideal for functional foods [12]. In addition, the seeds of common vetch could be used as a high-quality protein source [12,13]. Compared to soybeans and broad beans, common vetch is far more sensitive to soil salinity [4]. Salt stress can significantly reduce common vetch seed germination and slow seedling growth [3,14], restrict root and stem development, leading to a substantial decrease in plant biomass [3], and severely impairs yield [3,4].

Given the economic and ecological significance of common vetch, a thorough understanding of its salt tolerance mechanisms is crucial. However, such efforts have been limited, and a genome-wide association study (GWAS) to decipher the genetic basis of this complex trait has yet to be conducted. The large size, complexity, and high repetitive sequence content of common vetch genome has historically hindered the genetic studies and trait-mapping efforts [9,15], particularly for agronomic traits like plant architecture, stress tolerance, and quality [11,12]. A significant breakthrough occurred in 2022 with the publication of a draft genome sequence has dramatically enhanced the capacity to identify trait-associated loci or genes through association mapping and linkage mapping and establishing a crucial genomic foundation for advanced genetic research and trait enhancement. The salt tolerance of common vetch is governed by multiple minor-effect genes and quantitative trait loci (QTL) [16,17]. Due to the inherent complexity of plant salt tolerance mechanisms and their interaction with environmental factors, developing salt-tolerant cultivars through conventional breeding has been challenging [18]. As a result, QTL mapping combined with tightly linked molecular markers has emerged as an effective way to mitigate salt stress damage in breeding programs [19,20]. Traditional genetic analyses based on bi-parental populations are limited to examine bi-allelic gene variants, which significantly constrains the exploration of natural genetic variation. To overcome this limitation, GWAS have been developed as a powerful alternative approach [21]. Compared with linkage mapping, GWAS shows more time-efficient, cost-effective and eliminates the need to develop bi-parental mapping populations [22,23], and provides a more comprehensive representation of natural genetic diversity. These advantages made GWAS an valuable tool for investigating complex traits in wheat, maize, and soybean.

Identifying salt tolerance genes and applying closely linked markers can accelerate the development of salt-tolerant crops. Common vetch plants are sensitive to salt stress during the seedling stage compared to later growth phases, making common vetch seedling tolerance a critical research focus. However, genetic studies on salt tolerance in common vetch remain limited, and known genetic factors are insufficient for breeding applications. In this study, we used GWAS to analyze 5 salt tolerance traits across 172 common vetch varieties, aiming to (1) uncover the genetic basis of salt tolerance by GWAS, and (2) identify candidate genes for further studies.

## 2. Materials and Methods

### 2.1. Plant Materials and Treatments

**Figure 1:**
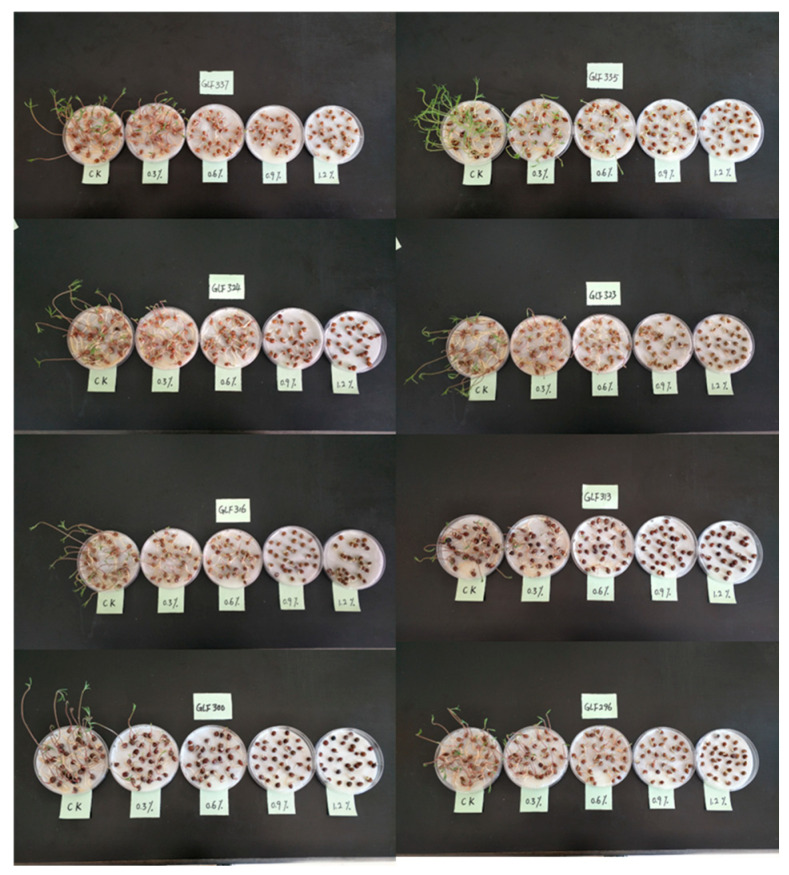
The performance of 8 different common vetch accessions under NaCl concentrations of 0.3%, 0.6%, 0.9%, and 1.2%.

A total of 172 common vetch accessions were collected from 18 countries used for the GWAS of the five salt tolerance-related traits. Most accessions originated from China, followed by Russia, Ukraine and Spain (Table A1, Figure 1). Chinese accessions were provided by the National Crop Germplasm Resources Green Manure Medium-term Storage Facility. Plump, uniformly sized seeds without insect damage were selected for seeding. Surface sterilization was performed by soaking seeds in 1% sodium hypochlorite (NaClO) for 10 min, followed by thorough rinsing under running tap water for 20 min. Based on previous reports, salinity stress was applied at 1.2% concentration using distilled water with an electrical conductivity (EC) of approximately 23.5 dS/m as control (CK). Double layers of filter paper were placed in 90 mm diameter Petri dishes and moistened with saline solution or distilled water until saturated without excess. Thirty surface-sterilized seeds were randomly placed in each dish. Both treatments (CK and salt) were replicated three times. Covered dishes were incubated in a constant temperature chamber at 25 °C.

### 2.2. Phenotypic Measurement and Data Analysis

To more accurately capture the salt tolerance variability among common vetch accessions at the germination stage while eliminating confounding effects attributable to inherent seed differences, 8 accessions were randomly selected from a panel of 172 germplasm resources and subjected to germination assays under a gradient of NaCl concentrations. Finally, 1.2% NaCl level markedly suppressed radicle growth and strongly inhibited germination, thereby providing an effective discriminatory threshold for salt tolerance. Under this concentration, accessions GLF337, GLF335, GLF323 and GLF316 retained full germinability, whereas GLF324, GLF313, G296 and GLF300 exhibited partial or complete loss of germination capacity (Figure 1). Thus, we selected a 1.2% NaCl level as the final stress level in this study, which also relatively close to the stress concentrations set in previous studies [4]. We evaluated the salt tolerance-related traits for all the 172 common vetch accessions under 1.2% NaCl level.

Germination was defined as radicle emergence exceeding 2 mm. Initial and final germination counts followed the Rules for Forage Seed Testing [24]. The following parameters were calculated: Germination rate (GR) = (seeds germinated by day 5/total seeds) × 100; Germination potential (GP) = (seeds germinated by day 14/total seeds) × 100; Germination index (GI) = Σ(Gt/Dt), where Gt = seeds germinated day, and Dt = days to germination. At final count, ten normal seedlings per replicate were randomly selected for measurements: root length (RL): average length from embryo to longest root tip; shoot height (SH): Average length from embryo to coleoptile/shoot tip; Basic statistical analyses for all traits under both treatments were performed using SAS v9.0.

Phenotypic data were subjected to outlier analysis using the interquartile range (IQR) method. Data points lying beyond 1.5 × IQR from the quartiles were considered outliers and excluded from subsequent analyses. Less than 2% of the data points were removed as outliers.

### 2.3. Re-Sequencing, Population Analysis and GWAS

To avoid the following issues with the collected common vetch seeds: (1) mixing of seeds from different varieties; (2) inherent instability of traits within the variety itself; and (3) genetic instability of the variety during germplasm collection, such as unstable traits in offspring and susceptibility to segregation, we conducted two years of seed multiplication and purification in Harbin, Heilongjiang. Unstable (segregating) and phenotypically heterozygous (in field and grain, etc.) varieties were removed, ultimately resulting in 172 common vetch accessions. This process ensured the purity and accuracy of the sequencing materials. For re-sequencing, 10 seedlings with consistent growth were selected, DNA was extracted from young leaves, and after concentration detection, equal amounts were mixed for resequencing.

Whole-genome re-sequencing of 172 accessions was conducted by PE150 sequencing, and yielding 3766.95 Gb raw data. After quality control, 3727.56 Gb clean data were retained. Clean reads were aligned to the reference genome (GCF_026540005.1) using the BWA (v0.7.8) [25]. Alignment achieved a 99.03% mapping rate. SNP calling was performed using GATK HaplotypeCaller (v4.0.10.1) [26] and were filtering by minor allele frequency (MAF < 0.05) and missing rate > 10% for further GWAS analysis. Population structure was analyzed using ADMIXTURE (v1.3.0) [27], whereas the principal component analysis (PCA) and neighbor-joining trees were constructed by TASSEL v5.0 [28]. GWAS employed a mixed linear model (MLM) in TASSEL v5.0, incorporating kinship matrices and principal components as covariates. In this study, the Bonferroni-Holm correction for multiple testing (alpha = 0.05) was too conservative, and only a few significant MTAs were detected. Therefore, markers with an adjusted −log_10_ (*p*-value) ≥ 3.0 were considered significantly associated. The Genomic Inflation Factor (λ) is calculated using the GEMMA (https://github.com/genetics-statistics/GEMMA/releases, accessed on Jun 6, 2025). The details related to the resequencing of 172 common vetch accessions, including sequencing library construction, quality control, alignment, SNP calling, and population structure analysis, have all been reported in detail in previous studies [1,2].

## 3. Results

### 3.1. Re-Sequencing and Population Structure Analysis of the Diverse Panel

**Figure A1:**
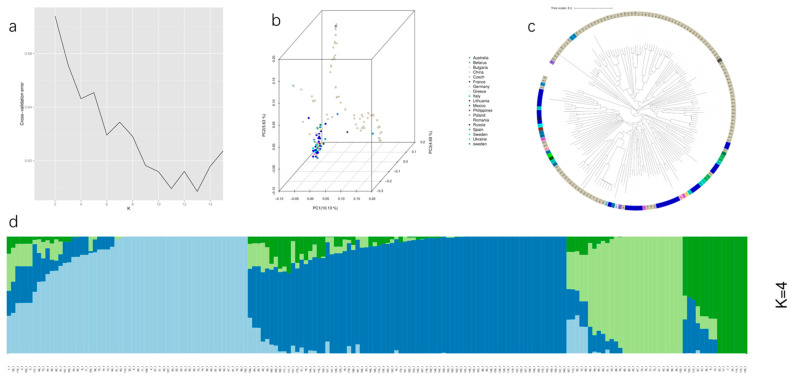
The population structure and PCA for the 172 common vetch accessions from Jin et al. [1,2]. (**a**): the population structure analysis (K = 4); (**b**): the PCA for the 172 common vetch accessions; (**c**): the neighbor-joining tree for the 172 common vetch accessions; (**d**): the population structure analysis for the 172 common vetch accessions.

Whole-genome re-sequencing of 172 common vetch accessions identified approximately 4.8 million high-quality SNPs distributed across the entire genome, with an average density of about 2900 SNPs/Mb. Collectively spanning over 1650 Mb, these SNPs revealed a non-uniform distribution of genetic variation across the genome. The re-sequencing data provide abundant and reliable SNP resources for GWAS and marker-assisted selection (MAS) breeding. We have reported the details of the SNP calling by Jin et al. [1,2]. The genetic architecture of 172 common vetch accessions, inferred from population structure analysis, resolved into four discrete subpopulations (K = 4; Pop1–Pop4). This subdivision was corroborated by principal component and phylogenetic analyses (Figure A1) [1,2]. The predominant subpopulation, Pop1 (n = 95), contained accessions from diverse regions including China, Russia, and Europe, suggesting a broad adaptive capacity. In comparison, Pop2 (n = 50) was almost exclusively Chinese, indicating localized genetic homogeneity. Pop3 (n = 15) and Pop4 (n = 12), while also predominantly Chinese, included rare accessions from Europe and Australia, hinting at genetically distinct lineages. Our findings reinforce previous classifications of Chinese common vetch into two or three major groups, wherein northwestern accessions show closer genetic ties to Europe, and southwestern populations possess unique adaptive characteristics. This geographically correlated genetic structure provides a foundational framework for regional breeding strategies.

### 3.2. Phenotype Analysis and GWAS of Salt Tolerance-Related Traits in Common Vetch

**Figure 2:**
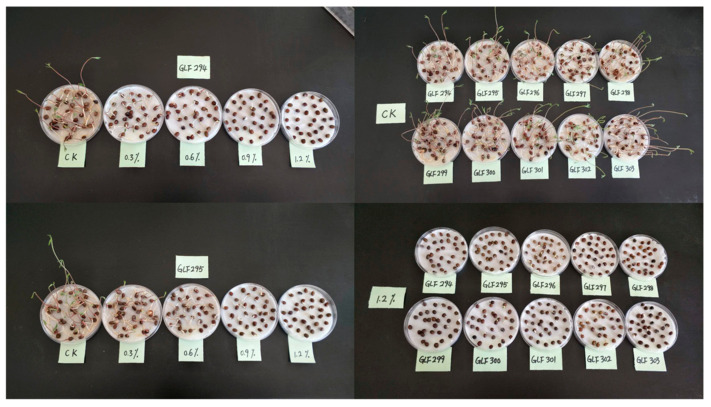
Phenotype analysis of the salt tolerance-related traits in common vetch accessions at 1.2% concentration level.

The phenotypic evaluation of five salt tolerance traits across 172 accessions (Table A2 and Table S1) revealed distinct patterns in trait variation. GI ranged from 16.40% to 49.40% (mean ± SE: 31.16% ± 5.81; CV = 18.64%), while GP showed broader variation (35.10-89.40%; 72.53% ± 11.58; CV = 15.96%). GR exhibited the least variation (62.20-96.80%; 85.22% ± 7.11; CV = 8.34%). In addition, the RL demonstrated the highest variability (1.50–7.40 cm; 4.04 ± 1.01 cm; CV = 25.04%), contrasting with SL, which showed the most consistent measurements (5.80–20.70 cm; 11.66 ± 1.64 cm; CV = 14.06%) (Table 1; Figure 2).

**Table 1:** Summary of the details of the 5 salt tolerance traits in common vetch.

	GI (%)	GP (%)	GR (%)	RL (cm)	SL (cm)
Minimum	16.40	35.10	62.20	1.50	5.80
Maximum	49.40	89.40	96.80	7.40	20.70
Mean	31.16	72.53	85.22	4.04	11.66
Standard error	5.81	11.58	7.11	1.01	1.64
Coefficient of variation	18.64%	15.96%	8.34%	25.04%	14.06%

GI: germination index; GP: germination potential; GR: germination ration; RL: Root length; SL: shoot length.

Correlation analysis indicated strong positive relationships among germination parameters, with GI showing very high correlation with GP (*r* = 0.924) and strong correlation with GR (*r* = 0.828). GR and GP were also strongly correlated (*r* = 0.854). Interestingly, RL displayed weak negative correlations with both GI (*r* = −0.383 and GP (*r* = −0.339), suggesting a potential trade-off between rapid germination and root development. SL exhibited moderate positive correlation with GR (r = 0.279) but only weak associations with other traits. The weakest observed correlation was between RL and GR (r = −0.026) (Table 2).

**Table 2:** Correlation coefficients among five traits of seedlings under salt stress.

	GI	GP	GR	RL
GP	0.924 *			
GR	0.828 *	0.854 *		
RL	−0.383 *	−0.339 *	−0.026	
SL	0.244 *	0.157 *	0.279 *	0.266 *

GI: germination index; GP: germination potential; GR: germination ration; RL: root length; SL: shoot length. * Correlation is significant at the 0.05 level.

**Figure 3:**
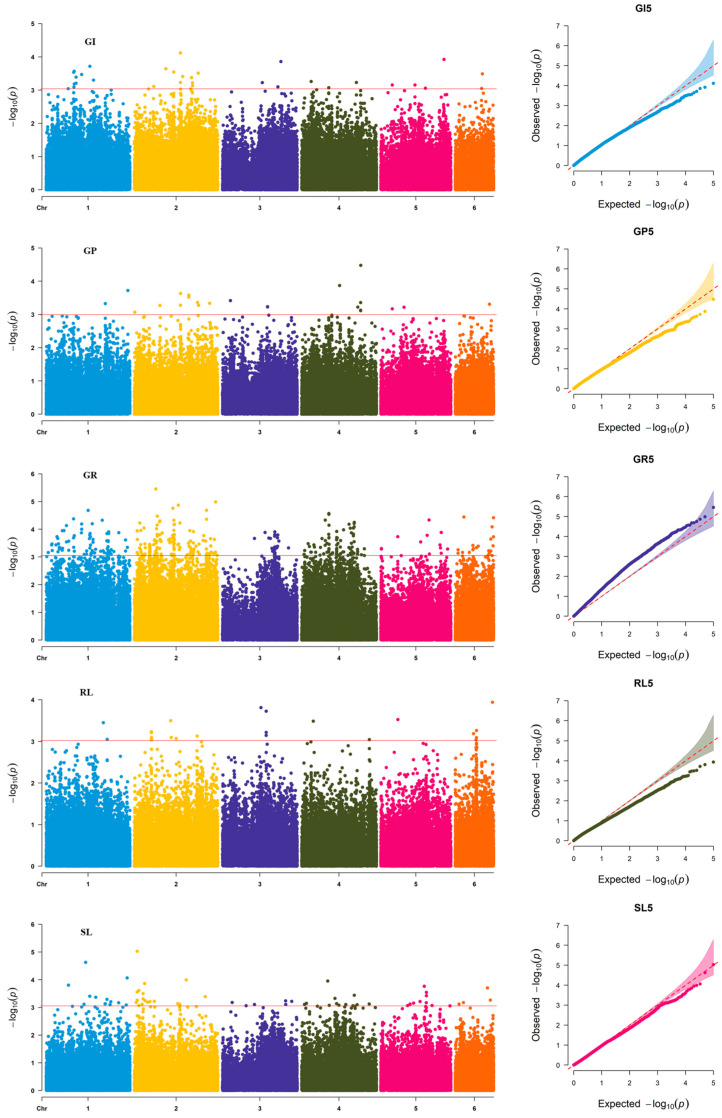
The Manhattan and Q-Q plot for salt tolerance traits in common vetch accessions. GI, germination index; GP, germination potential; GR, germination ratio; RL root length; SL, shoot length.

Totally, 20 loci significantly associated with salt tolerance across were identified (Table 3; Figure 3). The λGC values ranged from 1.02 to 1.08, indicating that our model effectively controlled for population structure and minimal inflation was present. Among these, chromosome 1 harbored four QTL: *qST1.1* (102.2–109.0 Mb) associated with SL, GR, and GI with PVE of 9.8–19.1%; *qST1.2* (137.6 Mb) related to SL and GI and explained PVE of 12.2–12.3%; *qST1.3* located at 221.5–229.6 Mb physical interval and influencing the RL, SL, and GP with explained the PVE of 11.5–14.5%; and *qST1.4* at the genetic interval of 310.7–316.8 Mb associated with SL and GP with PVE from 12.7% to 16.8%. Chromosome 2 contained 7 QTLs. The *qST2.1* located at 11.7 Mb, associated with SL and explained 12.0–21.8% of the PVE. The *qST2.2* at the genetic interval of 60.9–66.8 Mb for SL and RL with the PVE of 10.9–14.5%; *qST2.3* for GR and located at the genetic interval of 98.6–99.5 Mb with the PVE of 10.3–20.3%. *qST2.4* (153.1–155.2 Mb), related to GI and GR, and explained 14.5–20.3% of the PVE; *qST2.5* (176.0–179.4 Mb), linked to SL, GR, GI, and GP, accounted for 9.7–18.9% PVE; *qST2.6* (222.2–225.8 Mb), specific to GI, showed a PVE of 12.8–13.7%; *qST2.7* (241.0–248.9 Mb) influencing SL, RL, GP, and GI, and explained 10.1–14.1% of the PVE.

Chromosome 3 contained two significant QTLs: *qST3.1* (170.3 Mb), associated with RL with explained 13.8–16.4% PVE, and *qST3.2* (245.3–245.8 Mb), linked to SL, accounted for 13.6% PVE. On chromosome 4, *qST4.1* (105.4–107.4 Mb) was identified affecting GI, GR, and SL, with a PVE ranging from 13.4% to 21.6%, while *qST4.2* (228.6–229.2 Mb) for GP, explained 10.1–16.2% of the PVE. *qST5.1* (173.3–176.8 Mb) associated with GI and SL and showing a PVE of 9.7–14.8%. In addition, chromosome 6 harbored four QTLs: *qST6.1* (31.6–33.4 Mb) linked to SL and GR, and explained 13.3–21.2% PVE; *qST6.2* (80.4–82.3 Mb), associated with RL and accounted for 11.0–14.3% of the PVE; *qST6.3* (132.4–135.8 Mb) influencing GP and SL, and showed a PVE of 10.3–10.7%; and *qST6.4* (142.5–143.8 Mb), linked to GR and RL, and explained 14.3–17.5% PVE (Table 3; Figure 3).

### 3.3. Identification of Candidate Genes

In this study, 7 candidate genes associated with salt tolerance in common vetch were identified through gene annotation (Table 4). On chromosome 1, two genes were identified: *jg42199* (107.8 Mb for *qST1.1*), encoding zinc finger MYM-type protein 1, and *jg43393* (137.3 Mb for *qST1.2*), encoding ubiquitin-like domain-containing protein. On chromosome 2, *jg6460* (63.0 Mb for *qST2.2*) encoding a transcription factor bHLH155, and *jg9864* (242.5 Mb for *qST2.7*) encoding an auxin-responsive protein IAA8. On chromosome 3, *jg55573* (244.0 Mb for *qST3.2*) was identified and encoding a serine/threonine-protein kinase. Two genes were located on chromosome 6: *jg3633* (136.4 Mb for *qST6.3*) encoding an AP2-like ethylene-responsive transcription factor, and *jg4095* (144.9 Mb for *qST6.4*) encoding an ethylene-responsive transcription factor.

## 4. Discussion

Significant progress has been made in understanding the physiological and molecular mechanisms of salt tolerance in staple crops like rice, wheat and maize. Common vetch, an important green manure and forage crop that adapts well to various conditions, would greatly benefit from improved salt tolerance for better use of saline soils. However, current understanding of salt tolerance genes in common vetch remains limited [29], which constrains breeding progress. Further exploration and in-depth study of these loci are crucial for expanding vetch cultivation in saline areas. GWAS is an effective way for analyzing complex traits and can efficiently mapping genetic loci of salt tolerance and accelerating MAS breeding. The diverse panel applied in this study, composed of advanced lines and cultivars from diverse regions, exhibits rich genetic diversity. This provides valuable insights for breeding salt-tolerant common vetch [15,30]. Recent technological advancements, including the availability of a high-quality reference genome [9], high-depth re-sequencing data, genotyping arrays, and kompetitive allele-specific PCR (KASP) marker platforms, have significantly enhanced genetic research capabilities in crops. Our findings provide crucial insights into the genetic architecture of salt tolerance and establish a foundation for molecular breeding efforts aimed at expanding common vetch cultivation in salinity-prone regions.

In the present GWAS on salt tolerance-related traits in common vetch, which employed 4,796,342 markers, we observed a highly polygenic genetic architecture with generally small individual locus effects (∼11–18%). Several multiple-testing correction methods were explored, yet all yielded thresholds that proved overly stringent for our data. For instance, the Bonferroni–Holm method (α = 0.05) produced a threshold of *p* = 1.34 × 10^−8^, under which very few significant SNPs were detected. Similar outcomes were observed using the effective number of independent markers or false discovery rate controls—either few stable loci emerged, or none were detected. We attribute this stringency to two main factors: the genetic complexity of salt tolerance, involving numerous small-effect genes, and the extended LD decay (∼1.02 Mb) in common vetch, which reduces the effective number of independent tests. Thus, a simplified theoretical adjustment based on LD blocks suggests a threshold around *p* < 6.60 × 10^−4^, aligning with practices in other crops with complex genomes where thresholds of −log_10_ (*p*) ≥ 3.0 are commonly adopted.

The strong correlations among germination traits- GI, GP, and GR-indicate that salt stress similarly impacts both early and total germination in common vetch seedlings. The negative correlations between GI, GP and RL suggest a possible trade-off: rapid germination under salt stress might come at the expense of root development. Conversely, the positive correlation between GR and SL implies that higher germination rates support better shoot growth. However, the weak correlation between root and shoot traits (r = 0.227) indicates they respond somewhat independently to salt stress. These findings underscore the need to balance germination vigor and root development when breeding salt-tolerant common vetch.

Population structure analysis grouped the 172 vetch accessions into four subgroups (Pop1–Pop4), and characterization of the subgroups was largely consistent with geographic origins [1,2]. In addition, these results align with previous reports that Chinese vetch germplasm forms two to three distinct groups, with clear genetic differences from foreign materials [1,2,25,31]. Salt tolerance-related traits in common vetch are typically governed by quantitative inheritance and controlled by multiple minor genes. In this study, we identified 20 loci significantly associated with salt tolerance-related traits. Research on the genetic mechanisms underlying salt tolerance in common vetch has long been limited, with no clear reports on relevant genetic loci. Furthermore, this study utilized re-sequencing and derived SNP markers, which differ from traditional markers like SSR [15,23,25,29]. We also compared our findings with published transcriptomic and metabolomic studies on salt tolerance in common vetch but found no previously reported loci of genes consistent with our results. In addition, due to limited genetic studies on salt tolerance mechanisms in common vetch, there have been no reports to date on salt tolerance-associated genetic loci based on QTL mapping or genome-wide association studies (GWAS). We subsequently conducted comparative analyses with close relatives of common vetch, such as zombi pea (*Vigna vexillata*) and French pea (*Pisum sativum* L.). Laosatit et al. [32] reported two novel major QTLs, *qSaltol_3.1* and *qSaltol_7.1,* controlling seedling-stage salt tolerance were identified using a BC_1_F_2_ population derived from the salt-tolerant wild vetch accession “AusTRCF 322105” and the salt-sensitive line TVNu240. These QTLs explained 23–27% and 11–15% of the PVE for leaf wilting and plant survival, respectively. A genetic linkage map of zombi pea was constructed using an F_2_ population derived from a cross between salt-resistant JP235908 and salt-susceptible TVNu240, leading to the identification of three QTLs, *qSaltol1.1*, *qSaltol2.1*, and *qSaltol6.1*, associated with salt tolerance, with *qSaltol1.1* showing synteny to a known salt tolerance locus in beach cowpea and candidate genes encoding plasma membrane H^+^-ATPase and cation/proton exchanger implicating conserved mechanisms of salt resistance across Vigna species [33]. El-Esawi et al. [34] characterized 25 French pea accessions using fatty acid profiling and AFLP markers, revealing significant genetic diversity and identifying three AFLP markers associated with crude oil content, while also demonstrating that the more genetically diverse Nain Ordinaire cultivar exhibits greater salt tolerance than Elatius 3, with both genotypes showing enhanced salt stress responses when primed with 5-aminolevulinic acid (ALA). Due to the current lack of a reference genome suitable for synteny analysis and the predominant use of traditional molecular markers such as SSR and AFLP in previous studies, effective physical localization of the loci cannot yet be conducted. Furthermore, there have been no reports on the genetic dissection of salt-alkali tolerance in common vetch. Therefore, we speculate that the 20 loci identified in this study represent novel genetic loci associated with stress tolerance in common vetch.

We identified several loci with effects on multiple traits. For example, the *qST1.1* affects SL, GR, and GI, while *qST2.5* is associated with SL, GR, GI, and GP. This suggests salt stress broadly impacts common vetch seedlings, affecting various physiological and morphological traits simultaneously. Salt tolerance in common vetch therefore appears to be a comprehensive trait integrating multiple mechanisms, rather than controlled by a single gene or pathway. The identification of these QTLs provides valuable targets for MAS in breeding programs aiming at improving salt tolerance [15,30]. For instance, *qST2.5* and *qST6.4*, which explain significant PVE, could be prioritized for developing molecular markers. Future research should focus on fine-mapping these QTLs to identify candidate genes and validate their functions using functional genomics approaches. Integrating these findings with gene editing and genomic selection could accelerate the development of salt-tolerant common vetch varieties.

In this study, we have identified 7 candidate genes associated with salt tolerance in common vetch. Among these, MYM-type zinc finger proteins (*jg42199*) play a crucial role in plant salt-alkali tolerance by regulating downstream gene expression and interacting with stress response factors [35,36]. Its Arabidopsis ortholog, *AtZFP1*, has been functionally characterized to enhance salt stress tolerance. Overexpression of *AtZFP1* in Arabidopsis led to improved seed germination and root growth under salt stress, likely by modulating the expression of stress-responsive genes [37]. Similarly, ubiquitin-like proteins (*jg43393*) contribute to stress adaptation by modulating protein stability, subcellular localization, and function [38,39]. The Arabidopsis ortholog *AtDRM1*, which contains a ubiquitin-like domain, is involved in the regulation of seed germination and abiotic stress tolerance. Mutants of *AtDRM1* showed increased sensitivity to salt stress during post-germinative growth [40]. *jg6460* encodes a bHLH transcription factor, which may interact with DREB/CBF and NAC transcription factors to enhance salt tolerance [41,42]. In Arabidopsis, the bHLH transcription factor bHLH122 has been demonstrated to be a positive regulator of salt and drought tolerance. Transgenic plants overexpressing bHLH122 exhibited enhanced tolerance, whereas loss-of-function mutants were more sensitive, linking its function directly to abiotic stress signaling pathways [43]. *jg55573* encodes a serine/threonine-protein kinase involved in protein degradation and signal transduction; it activates or inhibits specific biological processes like osmotic regulation, antioxidant response, and ion balance [44,45]. An excellent example is the *Medicago truncatula* salt-tolerant genotype-specific gene *MtCIPK2*, a serine/threonine protein kinase. Functional analysis confirmed that *MtCIPK2* plays a critical role in conferring salt tolerance by regulating ion homeostasis and the antioxidant system [46]. Plant hormones play a central role in regulating growth and development [47,48]. *jg9864* encodes an auxin-responsive protein involved in root development and ion homeostasis, which are crucial for stress adaptation [7,49]. Auxin signaling is crucial for root architecture remodeling under stress. The Arabidopsis IAA8 protein itself has been implicated in this process. Studies indicate that the iaa8 mutant displays altered lateral root development and increased sensitivity to salt stress, suggesting that IAA8 plays a role in fine-tuning auxin responses to cope with ionic stress [50]. On chromosome 6, we identified two ethylene-responsive transcription factors, *jg3633* and *jg4095*. Ethylene and auxin synergistically regulate salt tolerance by optimizing stomatal closure, maintaining ion homeostasis, and reducing oxidative damage [51,52]. These genes belong to the well-known AP2/ERF superfamily. A key ortholog is the Arabidopsis *AtERF1* gene. Research has shown that *AtERF1* is rapidly induced by salt stress and acts as a central integrator of ethylene and jasmonate pathways, activating downstream defense genes and conferring improved salt tolerance [53]. Together, these candidate genes provide a comprehensive understanding of the genetic and molecular basis of salt tolerance in common vetch. Future research should focus on functionally validating these genes through transgenic studies and gene editing, as well as integrating them into breeding programs to develop resilient common vetch varieties for saline environments.

The observed negative correlations between key germination traits (GI, GP) and RL under salt stress suggest a significant physiological trade-off in the early seedling establishment of common vetch. We hypothesize that this trade-off reflects a fundamental resource allocation dilemma under stress. Accessions that prioritize rapid germination and shoot establishment (high GI/GP) may do so by allocating limited metabolic resources (e.g., carbohydrates, energy) and hormonal signals towards the embryo axis, potentially at the expense of subsequent radicle elongation and root system development. Conversely, genotypes that invest more in initial root growth might experience a delay in the mobilization of resources for cotyledon emergence and shoot expansion.

The identification of pleiotropic loci, such as *qST1.1* and *qST2.5*, which influence both germination/vigor traits and root/shoot growth, provides a genetic basis for this coordinated response. We propose several mechanistic pathways through which these loci might exert such disparate effects: (1) hormonal crosstalk as a central regulator. The candidate genes annotated within these pleiotropic QTL intervals are notably enriched for hormonal regulators. For instance, an auxin-responsive protein (IAA8, found near *qST2.7*) and ethylene-responsive transcription factors (near q*ST6.3* and *qST6.4*) are prime candidates. Both auxin and ethylene are master regulators of root architecture and are also deeply involved in seed germination (e.g., by counteracting the germination inhibitor ABA). A pleiotropic locus could encode a regulatory protein that modulates the sensitivity or distribution of these hormones. For example, a SNP could alter the expression of an auxin repressor, leading to simultaneously enhanced root elongation (via altered auxin signaling) and modified germination speed (via interaction with the ABA/GA balance), thereby directly linking the two processes. (2) resource mobilization and signaling hub. The serine/threonine-protein kinase (near *qST3.2*) represents another class of pleiotropic regulator. Such kinases often act as central hubs in stress signaling networks, integrating the salt stress signal and phosphorylating downstream targets that control diverse processes. This could include regulators of sugar metabolism or transport. The same kinase might simultaneously promote the breakdown of seed reserves to fuel germination (affecting GI/GR) and inhibit cell expansion in the root meristem under stress, manifesting as the observed negative correlation. (3) transcription factors with broad roles. The bHLH transcription factor (near *qST2.2*) could function as a higher-level integrator. bHLH proteins are known to regulate hundreds of downstream genes involved in cell division, elongation, and stress responses. A single mutation in such a transcription factor could differentially alter the expression of distinct gene sets-one network promoting germination efficiency and another concurrently suppressing root growth as part of a coordinated “survival-first” strategy under acute stress. In conclusion, the pleiotropic loci likely do not control these traits in isolation but rather function as key nodes in the genetic network that orchestrates the plant’s overall resource allocation and developmental priorities under salt stress. Rather than being an arbitrary correlation, the trade-off is probably a programmed, adaptive response. Future functional studies on the specific candidate genes within *qST1.1* and *qST2.5* will be crucial to validate these proposed mechanisms.

Also, this study still has many limitations. First, the GWAS was conducted using a panel of 172 common vetch accessions. Although this panel was carefully selected to encompass broad genetic diversity, originating from 18 countries, including the crop’s center of origin in Southern Europe and Western Asia, as well as key cultivation regions such as China and Russia, the sample size remains modest for dissecting a highly polygenic trait like salt tolerance. Moreover, the panel lacks representation from certain major regions, such as North and Latin America, which may limit the generalizability of the identified markers across all genetic backgrounds. To partially mitigate the constraints of sample size, we employed a high-density SNP set and a Mixed Linear Model that incorporated population structure and kinship, which helps enhance the reliability of the associations. Second, the candidate genes proposed in this study were inferred based on genomic annotations and orthologs involved in stress responses; however, these statistical associations have not yet been functionally validated. Complementary evidence from transcriptomic, physiological, or transgenic studies is required to confirm their causal roles. Third, the use of a suggestive significance threshold (−log_10_(*p*) ≥ 3.0) was adopted in light of the highly conservative nature of standard multiple-testing corrections for complex traits, an approach also utilized in other crop GWAS studies. While this facilitated the detection of loci with moderate effects, it underscores the polygenic architecture of salt tolerance and highlights the need for further validation. Future work will focus on expanding the germplasm collection, functionally characterizing candidate genes, and validating the pleiotropic QTLs in diverse genetic backgrounds to advance the breeding of salt-tolerant common vetch.

The identification of multiple QTLs and candidate genes associated with salt tolerance provides genetic resources for breeding. Several genomic regions had significant effects on key salt tolerance traits like shoot length, germination rate, and root development. Notably, *qST1.1* and *qST2.5* showed pleiotropic effects on multiple traits and explained substantial phenotypic variation. These findings suggest MAS targeting these regions could significantly enhance breeding efficiency. Breeding strategies should focus on pyramiding favorable alleles from these QTLs to achieve multi-trait improvements in salt tolerance. Furthermore, accessions carrying more favorable alleles and exhibiting superior salt tolerance traits alongside appropriate agronomic traits, such as HZMC1352, GLF303, GLF301, HZMC1387, GLF306, GLF368, GLF342, HZMC1384, HZMC1355, GLF307, and HZMC1366, are recommended as parental lines for improving salt tolerance.

## 5. Conclusions

This study successfully identified 20 novel loci and 7 candidate genes associated with salt tolerance in common vetch, providing critical insights into its genetic basis. The pleiotropic effects of 13 loci, such as *qST1.1*, *qST1.3*, *qST2.5*, and *qST2.7*, highlight their potential as key targets for breeding programs. Additionally, the discovery of candidate genes encoding regulatory proteins, including zinc finger MYM-type proteins and ethylene-responsive transcription factors, offers valuable molecular tools for enhancing salt tolerance. These findings not only deepen our understanding of salt tolerance mechanisms but also lay a foundation for developing resilient common vetch varieties, contributing to sustainable agriculture in saline-affected regions.

## Figures and Tables

**Table 3:** QTL mapping for salt tolerance identified in common vetch by GWAS.

QTL	Chromosome	Trait	Representative SNP Markers	Minimum Allele Frequency (MAF)	Physical Interval (Mb)	*p*-Value	R^2^ (%)
*qST1.1*	1	SL/GR/GI	102163780	0.23	102.2–109.0	9.20 × 10^−4^–4.23 × 10^−5^	9.8–19.1%
*qST1.2*	1	SL/GI	137568148	0.15	137.6–137.6	9.69 × 10^−4^–3.40 × 10^−4^	12.2–12.3%
*qST1.3*	1	RL/SL/GP	221538392	0.36	221.5–229.6	4.72 × 10^−4^–7.47 × 10^−4^	11.5–14.5%
*qST1.4*	1	SL/GP	310697884	0.35	310.7–316.8	8.15 × 10^−4^–8.61 × 10^−5^	12.7–16.8%
*qST2.1*	2	SL	11680009	0.32	11.7–11.7	5.54 × 10^−4^–9.43 × 10^−6^	12.0–21.8%
*qST2.2*	2	SL/RL	60914783	0.23	60.9–66.8	6.11 × 10^−4^–5.91 × 10^−4^	10.9–14.5%
*qST2.3*	2	GR	98579548	0.45	98.6–99.5	5.37 × 10^−4^–5.36 × 10^−5^	10.3–20.3%
*qST2.4*	2	GI/GR	153107319	0.36	153.1–155.2	2.86 × 10^−4^–4.72 × 10^−5^	14.5–20.3%
*qST2.5*	2	SL/GR/GI/GP	175986800	0.25	176.0–179.4	9.42 × 10^−4^–5.96 × 10^−5^	9.7–18.9%
*qST2.6*	2	GI	222197570	0.42	222.2–225.8	7.44 × 10^−4^–4.21 × 10^−4^	12.8–13.7%
*qST2.7*	2	SL/RL/GP/GI	241009562	0.42	241.0–248.9	7.39 × 10^−4^–4.35 × 10^−4^	10.1–14.1%
*qST3.1*	3	RL	170257249	0.29	170.3–170.3	6.05 × 10^−4^–1.88 × 10^−4^	13.8–16.4%
*qST3.2*	3	SL	245259620	0.32	245.3–245.8	7.80 × 10^−4^–5.81 × 10^−4^	13.6–13.6%
*qST4.1*	4	GI/GR/SL	105404419	0.36	105.4–107.4	8.45 × 10^−4^–2.68 × 10^−5^	13.4–21.6%
*qST4.2*	4	GP	228593912	0.28	228.6–229.2	7.68 × 10^−4^–3.32 × 10^−5^	10.1–16.2%
*qST5.1*	5	GI/SL	173258294	0.39	173.3–176.8	8.81 × 10^−4^–2.86 × 10^−4^	9.7–14.8%
*qST6.1*	6	SL/GR	31564891	0.25	31.6–33.4	6.71 × 10^−4^–3.63 × 10^−5^	13.3–21.2%
*qST6.2*	6	RL	80436698	0.36	80.4–82.3	9.12 × 10^−4^–5.44 × 10^−4^	11.0–14.3%
*qST6.3*	6	GP/SL	132399935	0.33	132.4–135.8	4.92 × 10^−4^–5.44 × 10^−4^	10.3–10.7%
*qST6.4*	6	GR/RL	142456787	0.28	142.5–143.8	1.15 × 10^−4^–8.16 × 10^−5^	14.3–17.5%

GI: germination index; GP: germination potential; GR: germination ration; RL: Root length; SL: shoot length.

**Table 4:** Identification of candidate genes in common vetch by GWAS.

Candidate Gene	Chr.	Physical Position (Mb)	QTL	Annotation
*jg42199*	1	107.8	*qST1.1*	Zinc finger MYM-type protein
*jg43393*	1	137.3	*qST1.2*	Ubiquitin-like domain-containing protein
*jg6460*	2	63.0	*qST2.2*	Transcription factor bHLH155
*jg3633*	6	136.4	*qST6.3*	AP2-like ethylene-responsive transcription factor
*jg4095*	6	144.9	*qST6.4*	Ethylene-responsive transcription factor
*jg9864*	2	242.5	*qST2.7*	Auxin-responsive protein IAA8
*jg55573*	3	244.0	*qST3.2*	Serine/threonine-protein kinase

GI: germination index; GP: germination potential; GR: germination ration; RL: root length; SL: shoot length.

## Data Availability

All datasets generated for this study are included in the article or supplementary material; further inquiries can be directed to the first author.

## Supplementary Materials

The following supporting information can be downloaded at: https://www.mdpi.com/article/10.3390/genes17010032/s1. Table S1: The salt tolerance related traits for common vetch under CK and 1.2% concentation NaCl.

## Abbreviations

The following abbreviations are used in this manuscript:

Abbreviation	Full name
GI	germination index
GP	germination potential
GR	germination ration
KASP	Kompetitive allele-specific PCR
MAS	Marker-assisted selection
PVE	Phenotypic variances explained
QTL	Quantitative trait loci
RL	Root length
SL	shoot length
SNP	Single-nucleotide polymorphism

## Appendix A

**Table A1:** The re-sequence details for the 172 common vetch accessions according to [1,2].

Accessions	Raw Base(bp)	Q_30_ (%)	Mapping Rate (%)	Average Depth
GLF294	21134455500	89.99	98.96	14.8
GLF295	19645967100	89.45	98.89	14.28
GLF295-1	20747940900	89.66	98.86	14.9
GLF296	21467760900	89.55	98.48	14.85
GLF297	20746617000	89.78	99.12	14.43
GLF298	21438588900	90.45	98.94	15.11
GLF299	21022150500	89.78	98.89	14.77
GLF300	20932939200	89.68	98.92	14.85
GLF300-1	20746878900	89.5	98.93	14.63
GLF301	21912388500	89.81	99.04	14.85
GLF302	20870989200	89.76	99.02	14.9
GLF303	20378051700	89.81	99.09	14.71
GLF303-1	20641681500	89.68	98.97	14.66
GLF304	21244262100	90	98.99	15.06
GLF304-1	19834464600	89.41	98.23	14.34
GLF305	21195542700	89.06	98.98	14.84
GLF306	20504720400	89.33	98.99	14.33
GLF307	21187016700	89.5	98.88	15.01
GLF308	21395083500	89.2	98.93	15.2
GLF309	20819532600	89.5	98.02	14.78
GLF309-1	20548575000	89.93	98.79	14.75
GLF310	20822583300	88.88	99.06	14.3
GLF310-1	20737351800	92.67	99.2	14.73
GLF311	19797642000	92.92	99.19	14.33
GLF312	23328246000	92.03	99.04	15.33
GLF313	22336798800	92.47	99.21	15.31
GLF314	19974556200	92.61	99.17	13.97
GLF315	24443194800	92.41	99.09	16.8
GLF315-1	21484123200	92.67	99.27	15
GLF316	22220646300	93	99.17	15.88
GLF317	24247050300	92.7	99.25	16.14
GLF318	20221660200	92.8	99.16	14.25
GLF318-1	21740703300	90.6	98.97	14.7
GLF319	22629246300	92.45	99.16	15.4
GLF321	22437187800	92.68	99.07	15.79
GLF322	21516501900	92.32	99.08	14.86
GLF323	20933554200	92.78	99.02	15.02
GLF323-1	22261429200	92.72	99.1	15.67
GLF324	22127114400	92.79	99.17	15.62
GLF325	22889834100	92.66	99.09	15.86
GLF326	21628465800	92.34	98.83	15.49
GLF328	21861675900	92.49	98.55	15.32
GLF329	22093706700	92.72	99.19	15.42
GLF330	21655981200	92.62	99.1	15.47
GLF331	21685816200	92.9	99.14	15.32
GLF332	21336471900	92.48	99.16	15.41
GLF333	22102731900	92.54	98.65	15.9
GLF333-1	21247605900	92.08	99.16	15.09
GLF334	21461705700	93.12	99.09	15.57
GLF329-1	22966098000	92.19	99.02	15.95
GLF335	36254537100	92.15	99.14	13.9
GLF336-1	21497134500	92.47	99.13	15.15
GLF336-2	21665347200	92.37	99.18	14.73
GLF337	21120644100	92.66	99.08	14.77
GLF338-1	21284688300	92.98	99.15	14.89
GLF338	22420985700	92.58	99.26	15.81
GLF339	19949240700	92.8	99.19	14.36
GLF340	20207098800	92.51	99.19	14.68
GLF340-1	21832117200	92.9	99.16	15.63
GLF341	21692525700	92.72	99.14	15.69
GLF341-1	21330411300	92.63	99.14	15.18
GLF342	21593575800	92.83	99.17	15.69
GLF342-2	22145169000	92.64	99.2	15.19
GLF343	20875257300	92.59	99.2	14.82
GLF344	20528463300	92.32	99.21	14.52
GLF345	22663639500	92.59	99.05	14.94
GLF346	23427512700	92.52	99.01	16.16
GLF347	24129963000	92.23	99.16	16.34
GLF347-2	21254089800	90.81	99.14	14.57
GLF347-1-1	23620626000	92.38	97.87	16.27
GLF347-1-2	24491873700	92.57	99.14	16.84
GLF348	22057065000	92.47	99.17	15.85
GLF349	20585715000	91.81	99.19	14.9
GLF349-1	19659573000	92.47	99.16	14.27
GLF349-2	21816666300	92.63	99.12	15.2
GLF350	22700386500	92.41	99.15	16.03
GLF350-2-1	30738479100	91.72	99.17	14.43
GLF350-2-2	26458402800	92.55	99.12	17.73
GLF351	21938719200	91.91	99.13	15.72
GLF352	22772430300	91.96	99.22	16.02
GLF353	21232386000	90.84	99.06	14.5
GLF354	24417186000	92.2	99.15	16.56
GLF355	23783206500	92.36	99.16	16.94
GLF356	21166077600	92.36	99.14	14.96
GLF357	19872339300	92.52	99.04	14.66
GLF358	21327718200	92.59	99.35	14.14
GLF359	19976148900	91.98	99.19	14.37
GLF360	21348723300	92.69	99.2	14.93
GLF361	20786276400	92.54	98.78	15.05
GLF362	20460021000	92.37	99.01	14.78
GLF363	21541807500	92.37	99.23	14.94
GLF364	21374652600	92.88	99.21	15.58
GLF364-2	23096432100	92.53	99.18	16.03
GLF365	20725632600	92.4	99.06	15.22
GLF366	24377565600	92.63	99.14	16.99
GLF367	21554040900	92.59	98.15	15.12
GLF368	21355528500	92.39	99.11	15.43
GLF368-2	20895023700	92.73	99.16	15.07
GLF369	21310533000	92.25	99.25	14.89
GLF370	20701425600	92.33	99.15	14.42
GLF371	19815053700	92.34	99.01	14.38
GLF371-1	21249645600	92.13	99.17	14.97
GLF373	21921892200	92.48	99.11	14.93
GLF373-2	24854070000	92.52	99.07	17.24
GLF529	21088897200	90.66	99.23	14.12
GLF530	21663326700	92.12	99.06	15.19
Longjian1	22466849700	92.09	99.14	15.72
Longjian2	20520950100	92.34	99.16	15.09
HZMC1349	24572042400	92.34	99.1	16.92
HZMC1349-2	20546892300	91.89	99.08	15.09
HZMC1350	20151500400	92.8	99.09	14.55
HZMC1352	20207601300	92.72	99.14	14.78
HZMC1353	21926967000	92.28	99.04	15.36
HZMC1353-2	21586321200	93.18	99.17	15.26
HZMC1354	25178001900	92.06	99.06	17.52
HZMC1355	28729092600	92.09	99.14	18.93
HZMC1356	23171207100	92.31	99.03	15.82
HZMC1357	22199913600	92.22	99.06	15.61
HZMC1358	21119421900	92.84	98.79	15.1
HZMC1362	22542300300	92.78	98.74	15.76
HZMC1362-1	20240970000	93.12	99.08	13.78
HZMC1363	23050270200	92.36	99.1	15.64
HZMC1364	20620193100	92.31	99.09	14.59
HZMC1364-1	20398821000	92.39	98.76	14.64
HZMC1365	21355562100	92.37	99.02	15.17
HZMC1366	22256711400	91.88	98.96	15.53
HZMC1366-2	20608162500	91.96	98.97	14.75
HZMC1367	20887590000	92.47	98.5	14.7
HZMC1368	24187035600	92.59	99.12	16.57
HZMC1369	22903100400	92.51	98.84	15.84
HZMC1370	20570032500	92.32	99.09	14.74
HZMC1371	24449571600	92.25	99.05	16.51
HZMC1372	20743684200	92.5	98.98	14.72
HZMC1374	19713392700	92.19	98.98	14.07
HZMC1375	20520146100	92.55	98.87	15.06
HZMC1376	21012910200	92.2	98.84	14.91
HZMC1377	22055028600	92.31	99.09	15.04
HZMC1378	22068342600	92.19	99.02	15.49
HZMC1379	23840644500	92.19	98.91	16.52
HZMC1380	22045690500	92.04	99.09	15.13
HZMC1381	27549707100	92.27	98.84	18.13
HZMC1382	21845141100	91.85	99.07	15.08
HZMC1382-1	20532626100	91.68	99.01	14.6
HZMC1382-2	20447736300	92.05	99.06	14.63
HZMC1383	20563463700	92.44	98.99	14.72
HZMC1383-2	21068715300	91.86	99.01	15.02
HZMC1384	21896156100	91.97	99.06	15.12
HZMC1385	20057544000	92.2	98.61	13.99
HZMC1386	21110608800	92.13	99.02	14.7
HZMC1387	38242274100	92.05	99.1	13.68
HZMC1388	23266070700	92.12	99.1	15.99
HZMC1389	20071114500	92.47	99.11	14.35
HZMC1477	20653504200	92.37	99.09	14.46
HZMC1479	21372073800	92.55	99.26	14.35
HZMC1480	21314790600	92.56	99.01	14.98
HZMC1481	20674823100	92.34	99.09	14.67
HZMC1482	20738642400	92.27	99.05	14.55
HZMC1483	19728652800	92.38	99.05	14.1
HZMC1484	21488101800	92.26	98.92	15.35
HZMC1485	25053127500	91.95	99.03	16.37
HZMC1486	20544990900	92.79	99.06	14.53
HZMC1487	21404729700	92.79	99.02	14.86
HZMC1488	21087528000	92.35	98.93	14.87
HZMC1489	21954798600	92.31	98.84	15.28
HZMC1490	21055472400	92.5	99.14	14.67
HZMC1491	21353447100	92.87	99.03	15.03
HZMC1492	20776690200	92.1	99.04	14.27
HZMC1493	21027942900	92.6	99.03	14.81
HZMC1494	21027960000	92.17	99.08	14.41
HZMC1535	21706517100	92.47	98.9	15.09
HZMC1535-1	20848357500	93.09	99.12	14.52
HZMC1536	20822493600	92.03	99.04	14.57

**Table A2:** The details of the 172 common vetch accessions and corresponding salt tolerance-related traits.

Trait	GI	GP	GR	RL	SL	Origin
GLF294	20.8	51.8	71.3	3.9	11.5	China
GLF295	32.2	75.6	92.1	5.3	11.6	China
GLF295-1	28.0	75.2	86.3	2.9	11.6	China
GLF296	41.0	86.3	94.9	2.9	12.2	China
GLF297	20.8	51.8	71.3	3.9	11.5	China
GLF298	25.9	61.6	80.4	5.3	11.8	China
GLF299	29.7	68.2	87.1	5.1	12.8	China
GLF300	32.8	78.9	88.9	4.0	11.8	China
GLF300-1	29.0	78.3	85.2	3.2	11.8	China
GLF301	29.3	68.2	88.3	4.0	11.8	China
GLF302	29.3	68.2	86.2	4.0	11.8	China
GLF303	41.0	85.5	89.9	2.9	12.2	China
GLF303-1	28.0	79.6	85.6	4.1	12.1	China
GLF304	41.0	89.1	94.6	2.9	12.2	China
GLF304-1	28.0	75.9	87.2	4.2	12.0	China
GLF305	41.0	89.1	92.7	2.9	12.2	China
GLF306	20.8	51.8	71.3	3.9	11.5	China
GLF307	29.3	68.2	82.0	4.0	11.8	China
GLF308	33.8	76.7	86.2	3.4	13.5	China
GLF309	29.7	68.2	89.6	5.1	12.8	China
GLF309-1	29.0	75.1	87.3	4.0	11.9	China
GLF310	41.0	89.1	92.1	2.9	12.2	China
GLF310-1	30.0	78.4	86.5	4.1	11.8	China
GLF311	24.4	55.3	75.6	4.6	9.3	China
GLF312	18.0	40.4	62.2	2.6	9.2	China
GLF313	29.7	68.2	88.0	5.1	12.8	China
GLF314	25.9	61.1	77.5	5.1	14.3	China
GLF315	30.4	73.3	85.1	4.3	12.2	China
GLF315-1	32.0	79.6	84.0	4.2	11.5	China
GLF316	41.0	89.1	91.2	2.9	12.2	China
GLF317	19.8	47.1	62.2	5.2	9.1	China
GLF318	32.8	77.7	83.6	4.0	11.8	China
GLF318-1	31.0	75.9	84.6	3.9	11.9	China
GLF319	41.0	89.1	93.8	2.9	12.2	China
GLF321	30.4	73.3	80.0	4.3	12.2	China
GLF322	41.0	89.1	92.3	2.9	12.2	China
GLF323	37.5	83.9	90.6	3.3	12.2	China
GLF323-1	30.0	78.3	85.6	4.2	11.7	China
GLF324	41.0	86.2	91.4	2.9	12.2	China
GLF325	32.2	75.6	88.7	5.3	11.6	China
GLF326	32.2	75.6	86.3	5.3	11.6	China
GLF328	30.4	73.3	85.1	4.3	12.2	China
GLF329	41.0	82.2	91.1	2.9	12.2	China
GLF330	41.0	89.1	90.7	2.9	12.2	China
GLF331	29.7	68.2	85.1	5.1	12.8	China
GLF332	20.8	51.8	71.3	3.9	11.5	China
GLF333	33.8	78.9	87.1	3.4	13.5	China
GLF333-1	32.0	80.2	87.3	4.1	11.8	China
GLF334	41.0	87.4	91.8	2.9	12.2	China
GLF329-1	31.0	81.2	87.2	4.0	11.5	China
GLF335	41.0	86.1	92.4	2.9	12.2	China
GLF336-1	20.8	51.8	71.3	3.9	11.5	China
GLF336-2	32.0	79.3	85.1	4.3	12.1	China
GLF337	41.0	89.1	92.2	2.9	12.2	China
GLF338-1	29.3	68.2	87.6	4.0	11.8	China
GLF338	33.0	84.2	85.9	3.9	12.0	China
GLF339	32.2	75.6	87.6	5.3	11.6	China
GLF340	36.4	83.8	92.1	5.4	11.8	China
GLF340-1	32.0	69.5	86.1	3.8	11.6	China
GLF341	32.8	78.9	86.8	4.0	11.8	China
GLF341-1	35.0	68.6	85.3	4.2	11.5	China
GLF342	32.2	75.6	93.8	5.3	11.6	China
GLF342-2	34.0	65.4	86.4	4.1	11.2	China
GLF343	33.8	78.9	87.1	3.4	13.5	China
GLF344	32.2	75.6	90.8	5.3	11.6	China
GLF345	30.4	73.3	85.1	4.3	12.2	China
GLF346	41.0	85.6	92.6	2.9	12.2	China
GLF347	33.8	78.9	86.1	3.4	13.5	China
GLF347-2	26.6	68.7	78.0	3.9	14.8	China
GLF347-1-1	30.0	64.6	86.3	4.0	11.7	China
GLF347-1-2	32.0	65.8	85.4	3.9	11.6	China
GLF348	32.2	75.6	89.3	5.3	11.6	China
GLF349	29.7	68.2	87.1	5.1	12.8	China
GLF349-1	29.0	70.2	85.2	4.2	11.7	China
GLF349-2	28.0	72.1	87.9	4.1	11.8	China
GLF350	41.0	87.2	85.0	2.9	12.2	China
GLF350-2-1	30.0	70.1	87.3	4.0	11.9	China
GLF350-2-2	31.0	70.3	86.2	4.3	11.6	China
GLF351	32.2	75.6	87.7	5.3	11.6	China
GLF352	29.3	68.2	87.4	4.0	11.8	China
GLF353	29.5	73.1	84.2	3.9	12.5	China
GLF354	32.2	75.6	92.8	5.3	11.6	China
GLF355	35.0	84.7	88.9	3.5	10.0	China
GLF356	25.9	61.6	80.4	5.3	11.8	China
GLF357	41.0	88.5	96.2	2.9	12.2	China
GLF358	32.8	78.9	88.9	4.0	11.8	China
GLF359	32.8	78.9	88.9	4.0	11.8	China
GLF360	32.8	78.9	85.6	4.0	11.8	China
GLF361	32.2	75.6	92.1	5.3	11.6	China
GLF362	29.3	68.2	87.4	4.0	11.8	China
GLF363	30.4	73.3	80.3	4.3	12.2	China
GLF364	25.9	61.6	80.4	5.3	11.8	China
GLF364-2	25.1	61.0	76.4	5.8	11.8	China
GLF365	32.2	75.6	85.5	5.3	11.6	China
GLF366	32.8	78.9	86.6	4.0	11.8	China
GLF367	21.6	52.0	70.7	4.4	10.2	China
GLF368	29.3	68.2	85.8	4.0	11.8	China
GLF368-2	24.4	55.7	80.2	4.2	11.3	China
GLF369	29.7	68.2	89.6	5.1	12.8	China
GLF370	29.7	68.2	89.6	5.1	12.8	China
GLF371	32.2	75.6	89.6	5.3	11.6	China
GLF371-1	33.0	78.0	90.0	5.4	11.2	China
GLF373	33.2	81.3	90.2	2.5	8.5	China
GLF373-2	34.7	81.8	91.4	3.2	8.4	China
GLF529	35.7	88.7	91.4	2.7	7.4	China
GLF530	36.8	89.4	91.6	2.2	6.4	China
Longjian1	33.8	78.9	87.1	3.4	13.5	China
Longjian2	30.8	65.5	79.1	4.5	20.7	China
HZMC1349	41.0	84.7	93.9	2.9	12.2	Russia
HZMC1349-2	38.1	75.7	86.8	4.0	19.4	Russia
HZMC1350	24.4	55.3	75.6	4.6	9.3	Russia
HZMC1352	32.8	78.9	85.8	4.0	11.8	Russia
HZMC1353	41.0	89.1	91.3	2.9	12.2	Russia
HZMC1353-2	49.4	88.0	96.8	2.1	14.0	Russia
HZMC1354	32.8	78.9	86.2	4.0	11.8	Russia
HZMC1355	41.0	85.5	91.7	2.9	12.2	Russia
HZMC1356	25.9	61.1	81.9	5.1	14.3	Russia
HZMC1357	32.2	75.6	91.0	5.3	11.6	Russia
HZMC1358	32.2	75.6	91.9	5.3	11.6	Russia
HZMC1362	33.2	81.3	90.2	2.5	8.5	Russia
HZMC1362-1	33.5	83.3	86.1	1.5	7.5	Russia
HZMC1363	32.8	78.9	88.9	4.0	11.8	Russia
HZMC1364	33.2	81.3	86.7	2.5	8.5	Russia
HZMC1364-1	33.6	83.4	84.4	1.6	8.4	Russia
HZMC1365	41.0	87.1	92.3	2.9	12.2	Russia
HZMC1366	32.2	75.6	89.1	5.3	11.6	Russia
HZMC1366-2	36.1	79.4	89.1	5.5	13.5	Russia
HZMC1367	32.2	75.6	89.5	5.3	11.6	Russia
HZMC1368	32.8	78.9	87.2	4.0	11.8	Russia
HZMC1369	32.2	75.6	90.7	5.3	11.6	Russia
HZMC1370	33.2	81.3	88.9	2.5	8.5	Russia
HZMC1371	32.2	75.6	91.2	5.3	11.6	Bulgaria
HZMC1372	32.8	78.9	88.3	4.0	11.8	Germany
HZMC1374	29.3	68.2	86.9	4.0	11.8	Greece
HZMC1375	29.3	68.2	86.8	4.0	11.8	Germany
HZMC1376	29.3	68.2	88.9	4.0	11.8	Russia
HZMC1377	27.5	70.4	87.9	4.3	11.2	Czech
HZMC1378	21.6	52.0	70.7	4.4	10.2	Sweden
HZMC1379	29.7	68.2	89.6	5.1	12.8	Russia
HZMC1380	33.8	78.9	87.1	3.4	13.5	Czech
HZMC1381	25.9	61.1	83.6	5.1	14.3	Poland
HZMC1382	25.9	61.6	80.4	5.3	11.8	Australia
HZMC1382-1	20.6	49.9	77.0	6.5	11.4	Australia
HZMC1382-2	16.6	41.2	73.7	7.4	10.5	Australia
HZMC1383	32.2	75.6	90.0	5.3	11.6	Spain
HZMC1383-2	21.7	52.5	82.6	7.3	10.1	Spain
HZMC1384	29.3	68.2	84.8	4.0	11.8	Spain
HZMC1385	20.8	51.8	71.3	3.9	11.5	Philippines
HZMC1386	25.9	61.6	80.4	5.3	11.8	Italy
HZMC1387	32.8	78.9	88.9	4.0	11.8	Ukraine
HZMC1388	32.8	78.9	87.0	4.0	11.8	Lithuania
HZMC1389	29.3	68.2	87.0	4.0	11.8	Russia
HZMC1477	32.8	76.9	88.3	3.4	11.9	Belarus
HZMC1479	25.5	59.8	64.0	1.9	5.8	Poland
HZMC1480	30.4	73.3	84.1	4.3	12.2	Sweden
HZMC1481	27.6	65.6	74.3	3.0	9.1	Belarus
HZMC1482	26.7	63.6	71.0	2.8	8.5	Belarus
HZMC1483	29.3	68.2	82.1	4.0	11.8	Ukraine
HZMC1484	27.4	65.0	75.9	3.4	9.7	Ukraine
HZMC1485	27.0	64.2	74.7	3.4	9.6	Ukraine
HZMC1486	29.3	68.2	85.1	4.0	11.8	Belarus
HZMC1487	18.0	40.4	62.2	2.6	9.2	Mexico
HZMC1488	19.0	42.1	68.7	3.0	10.3	Czech
HZMC1489	16.4	35.1	66.0	2.8	10.3	Spain
HZMC1490	18.0	40.4	62.2	2.6	9.2	Spain
HZMC1491	41.0	85.3	90.6	2.9	12.2	France
HZMC1492	29.5	73.1	81.1	3.9	12.5	Romania
HZMC1493	29.3	68.2	88.9	4.0	11.8	Ukraine
HZMC1494	29.7	68.2	89.6	5.1	12.8	Ukraine
HZMC1535	32.8	78.9	88.9	4.0	11.8	Russia
HZMC1535-1	32.0	78.3	89.1	4.2	11.5	Russia
HZMC1536	30.0	78.5	85.9	4.1	11.3	Russia

GI: germination index; GP: germination potential; GR: germination ration; RL: Root length; SL: shoot length.
